# Steroid-Induced Pancreatitis: A Challenging Diagnosis

**DOI:** 10.7759/cureus.8939

**Published:** 2020-07-01

**Authors:** Basma Ataallah, Mustafa Abdulrahman, Rana Al-Zakhari, Barjinder S Buttar, Shaha Nabeel

**Affiliations:** 1 Internal Medicine, Zucker School of Medicine at Mather, Port Jefferson, USA; 2 Internal Medicine, Northwell Health Mather Hospital, Port Jefferson, USA; 3 Internal Medicine, Flushing Hospital, Flushing, USA; 4 Internal Medicine, Richmond University Medical Center, Staten Island, USA; 5 Internal Medicine, Mather Hospital, Port Jefferson, USA

**Keywords:** corticosteroids, pancreatitis, drug induced pancreatitis

## Abstract

Drug-induced pancreatitis is uncommon among all cases of acute pancreatitis in the general population. The majority of reported cases are mild, but severe and even fatal cases have been also reported. Management of corticosteroid-induced acute pancreatitis requires withdrawal of the offending agent and supportive care.

Our case describes a young patient, who was recently diagnosed with idiopathic immune purpura and was treated with steroids. Few days later, he returned to the hospital complaining of epigastric pain with nausea and vomiting and was diagnosed with steroid-induced pancreatitis after exclusion of other causes of pancreatitis.

## Introduction

Drugs are a relatively rare cause of acute pancreatitis, with incidence rates between 0.1% and 2% [1]. Many drugs have been reported as the cause of the acute pancreatitis. Severity may vary from case to case, but usually is mild to moderate in severity and not associated with complications. Steroid-induced acute pancreatitis has been reported. However, the diagnosis of steroid-induced pancreatitis is challenging requiring careful review of the medical history and exclusion of other possible etiologies.

## Case presentation

**Figure 1:**
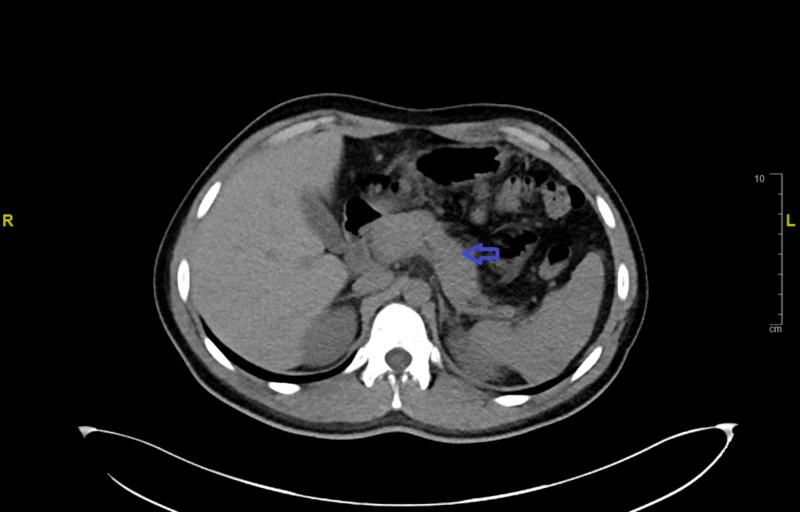
CT of the abdomen without intravenous or oral contrast showed acute pancreatitis (blue arrow)

A 20-year-old man with past medical history of asthma presented initially with bloody diarrhea and subjective fever of one-day duration. His vital signs were as follows: temperature 103°F, heart rate 110 beats/minute, blood pressure 110/70 mmHg, and respiratory rate 16 breaths/minute. On physical examination, there was generalized abdominal tenderness with normal bowel sounds without signs of rebound. Initial laboratory findings showed thrombocytopenia with platelet counts of 46 K/µl. Antibiotics were started for treatment of possible colitis due to associated fever and bloody diarrhea, but the platelet counts continued to trend down to 6 K/µl during the course of his inpatient hospitalization. The patient was diagnosed with idiopathic immune thrombocytopenia (ITP) secondary to viral infection versus toxic colitis. Thus, intravenous immunoglobulin and dexamethasone were administered. Platelet counts started to improve, and the patient clinically improved. He was discharged on oral prednisone. Three days later after discharge, the patient returned with epigastric pain with radiation to the back and associated nausea and vomiting. His lipase level was found to be elevated to 1,132 U/L and serum amylase was found to be 195 U/L. CT of the abdomen was performed and confirmed the diagnosis of acute pancreatitis (Figure 1). Other common causes of pancreatitis were excluded, no gallstones could be identified, both serum calcium and triglyceride levels were within the normal range, and the patient denied alcohol use. Steroid-induced pancreatitis was considered based on history, imaging study results, and biochemical markers. Prednisone was discontinued, and the patient improved after conservative management and was discharged home. 

## Discussion

Acute pancreatitis is a common cause of hospitalization in the United States. Biliary disease/gallstones and alcohol abuse account for 80% of cases. Other causes include: infections, trauma, scorpion bite, and other causes. Drug-induced pancreatitis accounts for 0.5%-2% of all cases [1]. Management of acute pancreatitis includes making the patient NPO, aggressive fluid replacement, and parenteral narcotic medications along with treating the underlying cause. 

The relationship between using high-dose steroid and acute pancreatitis has been previously reported in the literature. The diagnosis of such cases requires high suspicion, exclusion of other causes of pancreatitis, and a clear history of recent steroid use. Steroid-induced pancreatitis is a challenging diagnosis, which may require extensive workup to rule out other possibilities [1]. The mechanism by which oral glucocorticoid treatment might induce acute pancreatitis is unknown and needs to be investigated in future experimental studies; however, might be related to the alteration of lipid and calcium metabolism, the known systemic effect of corticosteroid; another mechanism found after injecting rabbits with steroids hypothesized that corticosteroids might obstruct small pancreatic ductules by leading to increased viscosity of pancreatic secretions, resulting in pancreatic changes. These changes included reduced basophilia, vacuolization of acini, peripancreatic fat necrosis, and hyperplasia of the islets of Langerhans [2]. Increasing doses of steroids may increase the risk of acute pancreatitis based on previous studies [3]. Generally, acute pancreatitis develops within 4-14 days of the initial exposure to the agent [4]. A careful review of the patients medications and their duration of use is required in cases of pancreatitis. If there is high suspicion for drug-induced pancreatitis, immediate discontinuation is required to prevent further damage to the pancreas [5]. 

Our case provides further evidence of the relationship between use of steroids and acute pancreatitis, even with short-term use of glucocorticoid administration.

## Conclusions

Health care providers should be able to identify a high-risk group that may be prone to corticosteroid-induced pancreatitis. Patients with previous history of pancreatitis and current or prior heavy alcohol users should be counseled about the association between the use of corticosteroids and the risk of pancreatitis. Identification of the offending agents is crucial to make the diagnosis of corticosteroid-induced pancreatitis. Treatment should focus on discontinuing corticosteroids and supportive care.
